# Associations of cigarette use, e-cigarette use, and dual use, with nocturia and urge urinary incontinence in US adults

**DOI:** 10.18332/tid/201399

**Published:** 2025-03-07

**Authors:** Xiangwei Yang, Yuhang Xie, Hong Chen, Junfu Zhang, Wenhan Qiu, Jun Pang

**Affiliations:** 1 Department of Urology, Kidney and Urology Center, Pelvic Floor Disorders Center, The Seventh Affiliated Hospital, Sun Yat-sen University, Shenzhen, China; 2 Guangming District Health Bureau, Shenzhen, China; 3 The Nethersole School of Nursing, Faculty of Medicine, The Chinese University of Hong Kong, Hong Kong SAR, China

**Keywords:** cigarette, e-cigarette, dual use, nocturia, urge urinary incontinence

## Abstract

**INTRODUCTION:**

Associations of cigarette use, e-cigarette use, and dual use, with nocturia and urge urinary incontinence (UUI) remain unclear. We performed this study to investigate the associations of cigarette use, e-cigarette use, and dual use, with nocturia and UUI.

**METHODS:**

This is a secondary analysis using data from the 2005 to 2020 National Health and Nutrition Examination Survey (NHANES). Participants aged ≥20 years were included. The use of cigarettes and e-cigarettes and symptoms of nocturia and UUI were self-reported. Logistic regression was used to calculate the adjusted odd ratios (AORs) of nocturia and UUI for cigarette use, e-cigarette use, and dual use. Subgroup analyses were conducted among participants with prostate cancer.

**RESULTS:**

Compared to never cigarette users, current and former cigarette users had higher odds of nocturia (AOR=1.12; 95% CI: 1.02–1.23; AOR=1.12; 95% CI: 1.01–1.24; all p<0.05) and UUI (AOR=1.23; 95% CI: 1.09–1.39; AOR=1.13; 95% CI: 1.03–1.24; all p<0.01). Compared to never e-cigarette users, current e-cigarette users had higher odds of nocturia (AOR=1.43; 95% CI: 1.01–2.02; p<0.05) and UUI (AOR=1.56; 95% CI: 1.13–2.16; p<0.01) and former e-cigarette users had higher odds of UUI (AOR=1.29; 95% CI: 1.02–1.62; p<0.05). Dual users of cigarettes and e-cigarettes had higher odds of nocturia (AOR=1.61; 95% CI: 1.03–2.51; p<0.05) and UUI (AOR=1.79; 95% CI: 1.19–2.68; p<0.01) compared to never users. In participants with prostate cancer, current cigarette use was associated with higher odds of UUI (AOR=2.40; 95% CI: 1.04–5.57; p<0.05.

**CONCLUSIONS:**

This study found that cigarette use, e-cigarette use, and dual use were associated with higher odds of nocturia and UUI. Cohort studies are needed to determine the causality of this cross-sectional assessment.

## INTRODUCTION

Nocturia and urge urinary incontinence (UUI) are among the most common lower urinary tract symptoms (LUTS) that significantly impact an individual’s quality of life^1^. Based on the International Continence Society, nocturia was defined as having woken to pass urine during the main sleep period for at least once, each urination must be followed by sleep or the intention to sleep, and UUI was defined as complaint of involuntary loss of urine associated with urgency^2^. Nocturia and UUI affect millions of men and women and their occurrence is highly associated with advanced age^3,4^. The prevalence of nocturia ranged from 11% to 35.2% in men and 20.4% to 43.9% in women aged 20–40 years, and from 68.9% to 93% in men and 74.1% to 77.1% in women aged ≥70 years^3^. The prevalence of UUI was 0.7% in men and 8.6% in women aged 18–39 years, 1.6% in men and 17.4% in women aged 40–59 years, and 7.3% in men and 32.8% in women aged ≥60 years^4^. Nocturia and UUI impair mental health, leading to anxiety and depression^5^, and increase risks of major adverse cardiac events^6^, falls, fractures, and death^7^. The economic burden of nocturia and UUI is substantial, with an estimated cost of $214.5 billion and $66 billion annually in the US, respectively, which is expected to increase as the population ages^4,8^.

Nocturia and UUI can be caused by many medical conditions, including prostatic diseases, cystitis, poorly controlled diabetes, renal insufficiency, and some neurologic disorders (e.g. Parkinson’s disease, pelvic or spinal nerve injury)^3,9^. Hypertension has been linked to a 1.2-fold to 1.3-fold risk of nocturia and a 2.1-fold risk of UUI^10,11^. Besides, several modifiable factors, such as sleep disorders^12^ and obesity^13^ are positively associated with nocturia and UUI. The impact of cigarette use on nocturia and UUI varies across different studies^13-18^. Although a questionnaire survey conducted in women showed no association between cigarette use and nocturia or UUI^14^, other studies have found that both male^15^ and female^16^ smokers had higher risks of nocturia and UUI than never smokers. Noh et al.^[Bibr CIT0017]17^ reported that cigarette use had negative impacts on all LUTS except nocturia. While in a multicenter study, Yoshimura et al.^[Bibr CIT0018]18^ found a negative association between cigarette use and nocturia. No study has reported the associations of e-cigarette use and dual use of cigarettes and e-cigarettes with nocturia and UUI.

Using data from the National Health and Nutrition Examination Survey (NHANES), we investigated the associations of cigarette use, e-cigarette use, and dual use with nocturia and UUI, among the general population. Additionally, as a secondary study aim, we explored the associations of cigarette use with nocturia and UUI in a subgroup of participants diagnosed with prostate cancer (PCa). Our hypotheses are that cigarette users, e-cigarette users, and dual users are at higher odds of nocturia and UUI compared to never users, and the associations between cigarette use and nocturia and UUI are robust in participants with PCa.

## METHODS

### Study population

This is a secondary dataset analysis of NHANES data. The NHANES is a nationally representative, cross-sectional survey administered by the National Center for Health Statistics. The survey assesses the health and nutritional status of the civilian non-institutionalized US population using a complex, multistage, probability sampling design, and purposely oversamples Hispanic, Black, Asian, and low-income individuals as well as the elderly population to provide more reliable and precise estimates of these groups. The National Center for Health Statistics Ethics Review Board approved the protocol, and all participants provided written informed consent. In this study, we analyzed data of the NHANES from 2005 to 2020 to examine the associations of cigarette use with nocturia and UUI, and data of the NHANES from 2015 to 2018 to examine the associations of e-cigarette use and dual use with nocturia and UUI. Participants were included in the analysis if they were aged ≥20 years. This age range was determined because target NHANES questions on nocturia and UUI were limited to participants aged at least 20 years. This study followed the Strengthening the Reporting of Observational Studies in Epidemiology (STROBE) reporting guidelines for cross-sectional studies.

### Assessment of cigarette use, e-cigarette use, and dual use

Cigarette use was assessed by the question: ‘Have you smoked at least 100 cigarettes in your entire life?’^13^. Individuals who answered ‘no’ were defined as never cigarette users, and those who answered ‘yes’ were asked whether they are smoking cigarettes currently by the question: ‘Do you now smoke cigarettes?’. Individuals with affirmative responses were defined as current cigarette users, and those who answered ‘no’ were defined as former cigarette users. E-cigarette use was assessed by the question: ‘Have you ever used an e-cigarette even one time?’. Individuals who answered ‘no’ were defined as never e-cigarette users and those who answered ‘yes’ were asked whether they are using it currently by the question: ‘During the past 30 days, on how many days did you use e-cigarettes?’. Individuals who answered ‘some days or everyday’ were defined as current e-cigarette users, and those who answered ‘no’ were defined as former e-cigarette users. Dual users were individuals who were both current cigarette users and current e-cigarette users. Exclusive cigarette users were individuals who were current cigarette users but not current e-cigarette users. Exclusive e-cigarette users were individuals who were current e-cigarette users but not current cigarette users. Never users were individuals who were never cigarette users and never e-cigarette users.

### Assessment of nocturia and urge urinary incontinence (UUI)

Nocturia was assessed by the question: ‘During the past 30 days, how many times per night did you most typically get up to urinate, from the time you went to bed at night until the time you got up in the morning?’. Individuals who answered once or more were defined as having nocturia. UUI was assessed by the question: ‘During the past 12 months, have you leaked or lost control of even a small amount of urine with an urge or pressure to urinate and you couldn’t get to the toilet fast enough?’ and individuals with affirmative answers were asked the frequency by the question: ‘How frequently does this occur?’. We quantified nocturia and UUI symptoms using a well-established questionnaire (Overactive Bladder Symptom Score)^[Bibr CIT0019]19^. Scores of 1, 2, and 3, were considered mild, moderate, and severe symptoms of nocturia or UUI, respectively.

### Ascertainment of prostate cancer (PCa)

Ascertainment of PCa was based on participant’s responses to the questions: ‘Have you ever been told by a doctor or other health professional that you had cancer or a malignancy of any kind?’ and ‘What kind of cancer?’. Persons with answers of ‘yes’ and ‘prostate cancer’ were assumed to have a diagnosis of PCa.

### Assessment of covariates

This study adjusted for several covariates. Age was categorized into three groups: 20 to 39 years, 40 to 59 years, and 60 years or older. Race was recoded into four categories: Hispanic (including Mexican American and other Hispanic), non-Hispanic White, non-Hispanic Black, and other races (which includes American Indian or Alaska Native, Native Hawaiian or Pacific Islander, multiple races or ethnicities, and those of unknown race). Education levels were grouped as follows: lower than high school, high school or equivalent, some college, and college graduate or above. Marital status was classified into three categories: married or living with partner, widowed, divorced, or separated, and never married. Family income was evaluated using the poverty-toincome ratio (PIR, a ratio that reflects annual family income relative to the federal poverty level) and categorized into low (PIR ≤1), middle (PIR 1–4), and high (PIR ≥4)^20^. Body mass index (BMI, kg/m^2^) was categorized into: normal (≥18.5 and <25), overweight (≥25 and <30), obese (≥30), and underweight (<18.5). Alcohol use was measured by the question: ‘During the past 12 months, about how often did you drink any type of alcoholic beverage?’. Individuals who drank every day or some days during the past 12 months were defined as drinkers, otherwise defined as non-drinkers. Sleep disorder was measured by the question: ‘Have you ever told a doctor or other health professional that you had trouble sleeping?’ and individuals with affirmative responses were deemed to have a sleep disorder. Diabetes was assessed by the question: ‘Have you ever been told by a doctor or other health professional that you had diabetes or sugar diabetes?’ and individuals with affirmative responses were defined as having a diabetes diagnosis, or defined by a hemoglobin A1c level of ≥6.5%^21^. Hypertension was assessed by the question: ‘Have you ever been told by a doctor or other health professional that you had hypertension, also called high blood pressure?’ and individuals with affirmative responses were defined as having a hypertension diagnosis, or defined by a measured systolic blood pressure of ≥140 mmHg, or a measured diastolic blood pressure of ≥90 mmHg^22^.

### Statistical analysis

Statistical analyses were performed using Stata 16.0 (StataCorp) from 21 February to 23 June 2024. Appropriate sample weights were constructed after combing survey cycles, and we used the Taylor series linearization method to calculate variance accounting for the complex survey design^8^. Participants’ characteristics were described by mean (standard deviation, SD) and frequencies (weighted percentages) and compared by cigarette use, e-cigarette use, and dual use using the chi-squared test and one-way analysis of variance as appropriate. Multivariable binary logistic regression was used to calculate the adjusted odds ratio (AOR) and 95% confidence interval (CI) of nocturia and UUI for cigarette use, e-cigarette use, and dual use. Additionally, we performed subgroup analyses stratified by gender and constructed multinomial logistic regression models to evaluate the impact of cigarette use on nocturia and UUI across different severity categories. The AOR and 95% CI of nocturia and UUI for cigarette use in participants with PCa were also calculated. All p-values were from 2-sided tests and results were deemed statistically significant at p<0.05.

## RESULTS

### Participant characteristics

**Figure 1:**
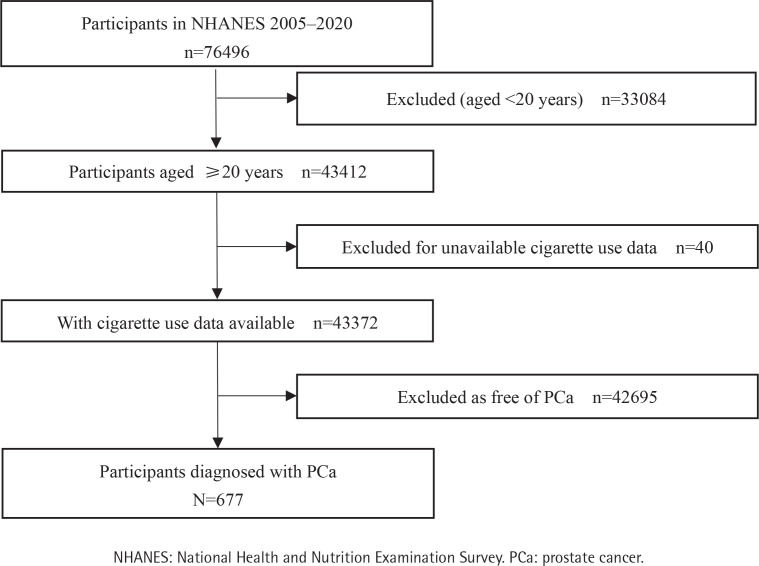
Study flowchart of participants in NHANES, 2005–2020 (N=76496)

**Figure 2:**
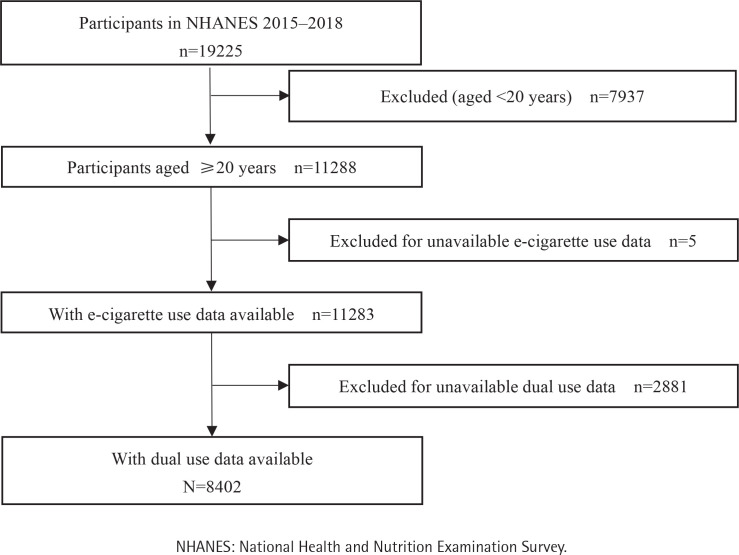
Study flowchart of participants in the NHANES 2015–2018 (N=19225)

Of the 76496 participants in NHANES 2005–2020, 43372 (56.7%) were aged ≥20 years and had information on cigarette use, among which 677 were diagnosed with PCa (Figure 1). Of the 19225 participants in NHANES 2015–2018, 11283 (58.7%) and 8402 (43.7%) were aged ≥20 years and had information on e-cigarette use and dual use, respectively (Figure 2). The mean (SD) age of the 43372, 11283, and 8402 participants was 47.5 (17.1) years (Table 1), 48.1 (17.3) years (Supplementary file Table 1), and 48.1 (17.3) years (Supplementary file Table 2), respectively. Table 1 shows that compared to never cigarette users, current and former cigarette users were more likely to be male, non-Hispanic White, have some college or lower education level, have a sleep disorder and hypertension (all p<0.001). Current cigarette users were less likely but former cigarette users were more likely to be older, married or living with a partner, have a middle-high family income, be overweight or obese, and have diabetes. Both current and former cigarette users were less likely to be drinkers (all p<0.001). Current and former e-cigarette users (Supplementary file Table 1) and dual users (Supplementary file Table 2) were more likely to be younger, male, have some college or lower education level, less likely to be married or living with a partner, and have a high family income (all p<0.001).

**Table 1:** Baseline characteristics of study participants by cigarette use^a^, NHANES 2005–2020 (N=43372)

*Characteristics*	*Cigarette use, n (weighted %)*				*p ^b^*
	*Total (N=43372)*	*Never (N=24222)*	*Current (N=8782)*	*Former (N=10368)*	
**Age** (years), mean (SD)^c^	47.5 (17.1)	46.0 (17.2)	43.1 (14.8)	54.2 (16.4)	<0.001
20–39	14569 (36.7)	9188 (40.4)	3532 (44.0)	1849 (22.5)	
40–59	14074 (37.0)	7781 (36.1)	3356 (41.1)	2937 (35.6)	
≥60	14729 (26.4)	7253 (23.5)	1894 (14.9)	5582 (41.9)	
**Gender**					<0.001
Male	21006 (48.1)	9675 (41.9)	5066 (54.5)	6265 (56.8)	
Female	22366 (51.9)	14547 (58.1)	3716 (45.5)	4103 (43.2)	
**Race**					<0.001
Hispanic	10771 (14.3)	6789 (16.7)	1660 (11.4)	2322 (11.1)	
Non-Hispanic White	17813 (66.4)	8411 (61.5)	4023 (67.7)	5379 (76.3)	
Non-Hispanic Black	9747 (11.4)	5548 (12.4)	2370 (14.0)	1829 (7.1)	
Other^d^	5041 (7.9)	3474 (9.4)	729 (6.9)	838 (5.6)	
**Education level**					<0.001
Lower than high school	10720 (15.9)	5374 (13.2)	2743 (23.8)	2603 (15.3)	
High school or equivalent	9993 (23.6)	4980 (20.4)	2585 (31.8)	2428 (24.1)	
Some college	12752 (31.1)	7010 (30.0)	2619 (32.4)	3123 (32.7)	
College graduate or higher	9852 (29.4)	6821 (36.4)	826 (12.0)	2205 (27.8)	
**Marital status**					<0.001
Married/living with partner	25489 (63.1)	14483 (64.2)	4438 (54.3)	6568 (67.8)	
Widowed/divorced/separated	9832 (18.6)	4853 (16.0)	2245 (22.6)	2734 (21.3)	
Never married	8017 (18.3)	4865 (19.8)	2093 (23.1)	1059 (10.9)	
**Family income**					<0.001
Low	8425 (14.4)	4185 (12.6)	2671 (25.4)	1569 (9.8)	
Middle	20562 (48.8)	11242 (46.9)	4141 (53.0)	5179 (49.7)	
High	9969 (36.7)	6187 (40.4)	1111 (21.6)	2671 (40.5)	
**BMI** (kg/m^2^)					<0.001
Normal	11030 (28.1)	6214 (28.6)	2710 (33.8)	2106 (22.6)	
Overweight	13413 (32.8)	7397 (32.5)	2568 (30.8)	3448 (35.1)	
Obese	15695 (37.5)	8876 (37.5)	2761 (32.4)	4058 (41.4)	
Underweight	656 (1.6)	300 (1.4)	266 (3.0)	90 (0.9)	
**Alcohol use**					<0.001
No	7171 (17.5)	3310 (15.5)	1351 (16.5)	2510 (22.2)	
Yes	25239 (82.5)	12949 (84.5)	6009 (83.5)	6281 (77.8)	
**Sleep disorder**					<0.001
No	32353 (72.5)	18998 (76.7)	6098 (67.4)	7257 (67.2)	
Yes	10992 (27.5)	5207 (23.3)	2680 (32.6)	3105 (32.8)	
**Diabetes**					<0.001
No	32844 (87.7)	18578 (88.9)	6916 (90.0)	7350 (83.2)	
Yes	6917 (12.3)	3534 (11.1)	1115 (10.0)	2268 (16.8)	
**Hypertension**					<0.001
No	21186 (59.5)	12607 (63.5)	4524 (61.2)	4055 (49.4)	
Yes	18649 (40.5)	9475 (36.5)	3546 (38.8)	5628 (50.6)	

NHANES: National Health and Nutrition Examination Survey. BMI: body mass index.

a Accounting for sampling weights.

b Calculated by chi-squared test or one-way analysis of variance.

c Individuals aged <20 years were not included in analyses due to a lack of information on nocturia and urge urinary incontinence.

d Other race includes American Indian or Alaska Native, Native Hawaiian or Pacific Islander, multiple races or ethnicities, or unknown.

### Associations of cigarette use, e-cigarette use, and dual use, with nocturia and urge urinary incontinence (UUI)

Table 2 shows that compared to never cigarette users, current cigarette users had higher odds of nocturia (AOR=1.12; 95% CI: 1.02–1.23; p<0.05) and UUI (AOR= 1.37; 95% CI: 1.23–1.52; p<0.001) in the model adjusted for age, gender, and race (Model 1) and higher odds of UUI in the model additionally adjusted for education level, marital status, and family income (Model 2) (AOR=1.23; 95% CI: 1.10–1.37; p<0.001) and in the model additionally adjusted for BMI, alcohol use, and sleep disorder (Model 3) (AOR=1.23; 95% CI: 1.09–1.39; p<0.01); former cigarette users had higher odds of nocturia (AOR=1.21; 95% CI: 1.10–1.33; p<0.001) and UUI (AOR=1.13; 95% CI: 1.03–1.24; p<0.01) in Model 1 and higher odds of nocturia in Model 2 (AOR=1.17; 95% CI: 1.06–1.29; p<0.01) and Model 3 (AOR=1.12; 95% CI: 1.01–1.24; p<0.05). Compared to never e-cigarette users, current e-cigarette users had higher odds of nocturia in Model 1 (AOR=1.44; 95% CI: 1.08–1.92; p<0.05) and Model 2 (AOR=1.43; 95% CI: 1.01–2.02; p<0.05) and higher odds of UUI in all models (all p<0.01); former e-cigarette users had higher odds of UUI in Model 1 (AOR=1.36; 95% CI: 1.07–1.72; p<0.05) and Model 2 (AOR=1.29; 95% CI: 1.02–1.62; p<0.05). Dual users had higher odds of nocturia in Model 1 (AOR=1.70; 95% CI: 1.11–2.59; p<0.05) and Model 2 (AOR=1.61; 95% CI: 1.03–2.51; p<0.05) compared to never users. Dual users, exclusive cigarette users, and exclusive e-cigarette users had higher odds of UUI in all models, compared to never users (all p<0.05).

**Table 2:** Associations of cigarette use, e-cigarette use, and dual use, with nocturia and urge urinary incontinence, NHANES 2005–2020

*Exposure*	*Nocturia, AOR (95% CI)*			*Urge urinary incontinence, AOR (95% CI)*		
	*Model 1*	*Model 2*	*Model 3*	*Model 1*	*Model 2*	*Model 3*
**Cigarette use**						
Never use ®	1	1	1	1	1	1
Current use	1.12 (1.02–1.23)*	1.00 (0.90–1.11)	0.99 (0.89–1.10)	1.37 (1.23–1.52)***	1.23 (1.10–1.37)***	1.23 (1.09–1.39)**
Former use	1.21 (1.10–1.33)***	1.17 (1.06–1.29)**	1.12 (1.01–1.24)*	1.13 (1.03–1.24)**	1.09 (0.99–1.19)	1.01 (0.92–1.12)
**E-cigarette use**						
Never use ®	1	1	1	1	1	1
Current use	1.44 (1.08–1.92)*	1.43 (1.01–2.02)*	1.39 (0.94–2.06)	1.72 (1.25–2.38)**	1.62 (1.18–2.23)**	1.56 (1.13–2.16)**
Former use	1.16 (0.96–1.39)	1.11 (0.92–1.33)	1.06 (0.88–1.28)	1.36 (1.07–1.72)*	1.29 (1.02–1.62)*	1.23 (0.98–1.55)
**Dual use**						
Never use ®	1	1	1	1	1	1
Dual use	1.70 (1.11–2.59)*	1.61 (1.03–2.51)*	1.57 (0.97–2.54)	1.98 (1.30–3.00)**	1.82 (1.25–2.65)**	1.79 (1.19–2.68)**
Exclusive cigarette use	1.23 (0.93–1.63)	1.08 (0.79–1.46)	1.06 (0.78–1.44)	1.54 (1.21–1.97)**	1.43 (1.08–1.90)*	1.46 (1.10–1.93)*
Exclusive e-cigarette use	1.34 (0.89–2.02)	1.35 (0.83–2.20)	1.31 (0.74–2.30)	1.76 (1.02–3.02)*	1.83 (1.02–3.29)*	1.77 (1.00–3.12)*

NHANES: National Health and Nutrition Examination Survey. AOR: adjusted odds ratio. Model 1: adjusted for age, gender, race. Model 2: adjusted as for Model 1 plus education level, marital status, and family income. Model 3: adjusted as for Model 2 plus body mass index, alcohol use, and sleep disorders. ® Reference categories.

* p<0.05,

** p<0.01,

*** p<0.001.

Subgroup analyses stratified by gender demonstrated that, compared to never users, current and former male cigarette users, as well as current male e-cigarette users, had higher odds of nocturia and UUI after adjusting for covariates (Supplementary file Table 3, all p<0.05). In females, former cigarette users had increased odds of nocturia (AOR=1.14; 95% CI: 1.00–1.31; p<0.05) compared to never cigarette users, adjusting for age and race (Supplementary file Table 4). Current cigarette users, current and former e-cigarette users, dual users, and exclusive cigarette users showed higher odds of UUI compared to never users after adjustment for covariates (Supplementary file Table 4, all p<0.05). Multinominal logistic regressions showed that former cigarette users had higher odds of mild (AOR=1.12; 95% CI: 1.01–1.25; p<0.05), moderate (AOR=1.33; 95% CI: 1.18–1.49; p<0.001), and severe (AOR=1.49; 95% CI: 1.30–1.71; p<0.001) nocturia compared to never cigarette users. Current cigarette users had higher odds of moderate (AOR=1.27; 95% CI: 1.11–1.47; p<0.01) and severe (AOR=1.67; 95% CI: 1.45–1.89; p<0.001) nocturia compared to never cigarette users, adjusting for age, gender, and race. In terms of UUI, compared to never cigarette users, current cigarette users had increased odds of mild (AOR=1.34; 95% CI: 1.21–1.49; p<0.001), moderate (AOR=1.54; 95% CI: 1.23–1.94; p<0.001), and severe (AOR=1.40; 95% CI: 1.09–1.80; p<0.01) UUI. Former cigarette users had higher odds of mild (AOR=1.11; 95% CI: 1.01–1.22; p<0.05) and moderate (AOR=1.29; 95% CI: 1.06–1.58; p<0.001) UUI compared to never cigarette users, after adjusting for age, gender, and race.

### Associations of cigarette use with nocturia and urge urinary incontinence (UUI) in participants with prostate cancer (PCa)

Table 3 shows that neither current cigarette users nor former cigarette users showed higher odds of nocturia than never cigarette users (all p>0.05). Current cigarette users had higher odds of UUI than never cigarette users in models adjusted for age (AOR= 2.33; 95% CI: 1.05–5.17; p<0.05), additionally adjusted for race, education level, marital status, and family income (AOR=2.40; 95% CI: 1.04–5.57; p<0.05), and additionally adjusted for BMI, diabetes, and hypertension (AOR=2.68; 95% CI: 1.05–6.83; p<0.05). Former cigarette users showed no higher odds of UUI compared to never cigarette users in all adjusted models (all p>0.05).

**Table 3:** Associations of cigarette use with nocturia and urge urinary incontinence in participants with prostate cancer, NHANES 2005–2020

*Exposure*	*Nocturia, AOR (95% CI)*			*Urge urinary incontinence, AOR (95% CI)*		
	*Model 1*	*Model 2*	*Model 3*	*Model 1*	*Model 2*	*Model 3*
**Cigarette use**						
Never use ®	1	1	1	1	1	1
Current use	0.96 (0.30–3.10)	1.45 (0.30–6.93)	1.23 (0.24–6.29)	2.33 (1.05–5.17)*	2.40 (1.04–5.57)*	2.68 (1.05–6.83)*
Former use	0.57 (0.32–1.02)	0.58 (0.31–1.09)	0.60 (0.31–1.19)	1.03 (0.63–1.66)	1.05 (0.64–1.74)	1.05 (0.63–1.76)

NHANES: National Health and Nutrition Examination Survey. AOR: adjusted odds ratio. Model 1: adjusted for age. Model 2: adjusted as for Model 1 plus race, education level, marital status, and family income. Model 3: adjusted as for Model 2 plus body mass index, diabetes, and hypertension. ® Reference category.

* p<0.05.

## DISCUSSION

Our study found that current and former cigarette use were associated with higher odds of nocturia and UUI compared to never cigarette use, especially for cases of moderate and severe nocturia and UUI. We first reported that current e-cigarette use and dual use were associated with higher odds of nocturia and UUI, and former e-cigarette use was associated with higher odds of UUI. In males, both current and former cigarette users, as well as current e-cigarette users, had higher odds of nocturia and UUI. In females, former cigarette users showed increased odds of nocturia, and current cigarette users, current and former e-cigarette users, and dual users had higher odds of UUI. Among participants diagnosed with PCa, current cigarette use was associated with higher odds of UUI.

Several mechanisms are responsible for the impact of cigarette use, e-cigarette use, and dual use on symptoms of nocturia and UUI. A typical cigarette contains approximately 10–15 mg of nicotine, nearly 10% of which is absorbed into the systemic circulation^23^. Nicotine binds to neuronal nicotinic acetylcholine receptors and modulates neurotransmitter release, leading to an increase of sympathetic nervous system activity that causes detrusor overactivity (DO) manifested as nocturia and/or UUI^13,24^. Nicotine is associated with elevated testosterone levels^24^, which result in benign prostatic hyperplasia (BPH) and bladder dysfunction that can also manifest as nocturia and/or UUI. Cigarette use causes atherosclerosis^25^, the presence of which in the pelvic vessels induces chronic bladder ischemia and results in DO^26^. Cigarette use increases risks of hypertension and diabetes^27,28^, causing sympathetic overactivity, atherosclerosis, hyperglycemia-associated BPH, increased calcium within the detrusor^24^ and leading to increased risks of nocturia and UUI^3,10,11^. Cigarette use is positively associated with obesity^29^, which causes LUTS by promoting aromatization of testosterone into estrogen, production of pro-inflammatory cytokines, and increased sympathetic activity^24^. Most e-cigarettes contain nicotine, and e-cigarette use also increases risks of atherosclerosis^30^, hypertension^31^, diabetes^32^, and obesity^33^ that contribute to nocturia and UUI^24^. Besides, the use of e-cigarettes produces heavy metals, several unique chemicals, including volatile organic compounds and ultrafine particles, and other unknown constituents^34^, which may play essential roles in causing nocturia and UUI, and further investigations are warranted. Dual use of cigarettes and e-cigarettes adds together the impact of both cigarette and e-cigarette use and theoretically increases the risks of nocturia and UUI. Prostate cancer is a common cause of nocturia and UUI^3,[Bibr CIT0009]9^. Current cigarette use in participants with PCa was associated with increased odds of UUI, suggesting that the impact of cigarette use on LUTS is independent of patients’ comorbidities.

Cigarette smoking accounted for the highest proportion of cancer cases (19.0%) and deaths (28.8%) and was responsible for the majority of deaths from heart diseases, chronic obstructive pulmonary diseases (COPD), and cerebrovascular diseases^35^, all of which were the most common causes of death in the US. Although the use of cigarettes has declined in recent years, there were still 34.2 million current cigarette users in 2018, accounting for 13.7% of US adults^36^. E-cigarettes have been considered to be less harmful than combustible cigarettes, but their long-term safety profile has not been determined. Increasing evidence has shown that e-cigarette use is also associated with increased risks of asthma, COPD, emphysema, and chronic bronchitis^34^. Furthermore, the prevalence of use of e-cigarettes has increased dramatically since their introduction to the US market^34^. Measures to prevent cigarette and e-cigarette smoking are essential to reduce smoking-attributable diseases and beneficial to decrease odds of nocturia and UUI.

### Strengths and limitations

Major strengths of this study include the use of a nationally representative survey, the implementation of subgroup analyses, and the adjustment for potential confounding factors, implying that our findings have excellent robustness and generalization. This study also has several limitations. The results are based on cross-sectional data and should be interpreted with caution. The use of self-reported questionnaires to assess both exposures and outcomes may lead to recall bias and misreporting. Additionally, we did not evaluate the impact of the number of cigarettes and/ or e-cigarettes smoked on nocturia and UUI. We also cannot rule out the possibility of residual confounding in our findings. Furthermore, the findings of this study among US adults may not be generalizable to other countries. Lastly, the sample size of participants with PCa is relatively small, and further validation of the results is necessary.

## CONCLUSIONS

Our study found significant positive associations of cigarette use, e-cigarette use, and dual use with nocturia and UUI. Cohort studies are needed to establish causality given the cross-sectional design of this study.

## Supplementary Material



## Data Availability

The data supporting this research are publicly available at https://wwwn.cdc.gov/nchs/nhanes/Default.aspx
