# Supplementary material for: Associations of cigarette use, e-cigarette use, and dual use, with nocturia and urge urinary incontinence in US adults
Source: Tob Induc Dis. 2025 Mar 7;23:10.18332/tid/201399. doi: 10.18332/tid/201399 (PMC11887039; doi:10.18332/tid/201399)
Supplement: Supplementary file 1 [file TID-23-25-s1.pdf]

Supplementary Table 1. Baseline characteristics of study participants in the NHANES from 2015 to 2018 by e-cigarette use[a] (N=11283)

Characteristic	E-cigarette use, No. (weighted %)				P value[b]
	Total (n=11283)	Never (n=9255)	Current (n=516)	Former (n=1512)	
**Age, mean (SD), years[c]**	48.1 (17.3)	50.8 (17.4)	37.2 (13.1)	38.9 (13.1)	<0.001
20-39	3638 (36.2)	2486 (29.7)	322 (62.3)	830 (58.4)	
40-59	3596 (35.3)	2998 (36.7)	141 (28.2)	457 (31.0)	
≥60	4049 (28.6)	3771 (33.6)	53 (9.4)	225 (10.6)	
**Gender**					<0.001
Male	5446 (48.1)	4312 (46.0)	299 (59.9)	835 (54.0)	
Female	5837 (51.9)	4943 (54.0)	217 (40.1)	677 (46.0)	
**Race**					0.052
Hispanic	3015 (15.5)	2597 (16.1)	105 (13.4)	313 (13.6)	
Non-Hispanic White	3795 (63.0)	2931 (62.0)	225 (66.4)	639 (66.8)	
Non-Hispanic Black	2494 (11.4)	2039 (11.6)	108 (9.9)	347 (11.0)	
Other[d]	1979 (10.0)	1688 (10.3)	78 (10.3)	213 (8.5)	
**Education level**					<0.001
Lower than high school	2479 (12.9)	2126 (13.0)	106 (18.2)	247 (10.4)	
High school or equivalent	2561 (23.9)	1995 (21.9)	149 (33.9)	417 (30.4)	
Some college	3468 (31.6)	2660 (29.7)	215 (36.9)	593 (38.7)	
College graduate or above	2758 (31.6)	2457 (35.3)	46 (11.0)	255 (20.5)	
**Marital status**					<0.001
Married/living with partner	6691 (63.2)	5685 (66.0)	244 (48.4)	762 (54.4)	
Widowed/divorced/separated	2531 (18.4)	2144 (19.0)	95 (17.1)	292 (16.0)	
Never married	2053 (18.4)	1419 (15.0)	177 (34.6)	457 (29.7)	
**Family income**					<0.001
Low income	2058 (13.7)	1609 (12.2)	118 (19.9)	331 (18.9)	
Middle income	5371 (48.8)	4360 (47.6)	265 (57.2)	746 (51.6)	
High income	2429 (37.5)	2088 (40.1)	71 (22.9)	270 (29.5)	
**BMI category**					<0.001
Normal	2663 (25.8)	2146 (25.5)	136 (28.0)	381 (26.5)	
Overweight	3400 (31.5)	2880 (32.7)	136 (28.4)	384 (26.5)	
Obesity	4358 (41.3)	3523 (40.6)	196 (39.5)	639 (45.2)	
Underweight	156 (1.5)	115 (1.2)	17 (4.1)	24 (1.8)	
**Alcohol use**					<0.001
No	1867 (17.1)	1604 (18.2)	52 (10.9)	211 (14.4)	
Yes	6590 (82.9)	5064 (81.8)	401 (89.1)	1125 (85.6)	
**Sleep disorder**					<0.001
No	8193 (69.6)	6879 (71.6)	340 (62.6)	974 (62.0)	
Yes	3081 (30.4)	2369 (28.4)	176 (37.4)	536 (38.0)	
**Diabetes**					<0.001
No	8277 (86.0)	6641 (84.8)	412 (90.0)	1224 (90.7)	

Yes	2063 (14.0)	1839 (15.2)	51 (10.0)	173 (9.3)	
**Hypertension**					<0.001
No	5355 (58.8)	4181 (56.9)	297 (65.4)	877 (65.9)	
Yes	5026 (41.2)	4317 (43.1)	178 (34.6)	531 (34.1)	

NHANES, National Health and Nutrition Examination Survey; SD, standard deviation; BMI, body mass index.

[a] Accounting for sampling weights.

[b] Calculated by Chi-square test or one-way analysis of variance.

[c] Individuals under 20 years old were not included in analyses due to a lack of information on nocturia and urge urinary incontinence.

[d] Other race includes American Indian or Alaska Native, Native Hawaiian or Pacific Islander, multiple races or ethnicities, or unknown.

Supplementary Table 2. Baseline characteristics of study participants in the NHANES from 2015 to 2018 by dual use of cigarettes and e-cigarettes[a] (N=8402)

Characteristic	Dual use of cigarettes and e-cigarettes, No. (weighted %)					P value[b]
	Total (n=8402)	Never use (n=6115)	Dual use (n=302)	Exclusive cigarette use (n=1771)	Exclusive e-cigarette use (n=214)	
**Age, mean (SD), years[c]**	48.1 (17.3)	48.1 (17.1)	40.7 (12.8)	45.3 (15.5)	32.5 (12.1)	<0.001
20-39	2939 (38.8)	2009 (35.6)	164 (53.1)	608 (41.0)	158 (74.8)	
40-59	2875 (36.8)	2084 (37.1)	106 (34.7)	650 (39.0)	35 (19.5)	
≥60	2588 (24.4)	2022 (27.2)	32 (12.3)	513 (20.0)	21 (5.6)	
**Gender**						<0.001
Male	3676 (44.1)	2326 (39.7)	170 (57.2)	1051 (53.8)	129 (63.4)	
Female	4726 (55.9)	3789 (60.3)	132 (42.8)	720 (46.2)	85 (36.6)	
**Race**						0.002
Hispanic	2254 (16.1)	1798 (17.3)	52 (11.3)	351 (12.7)	53 (16.3)	
Non-Hispanic White	2587 (60.5)	1690 (59.3)	140 (67.1)	672 (62.5)	85 (65.5)	
Non-Hispanic Black	1947 (12.6)	1313 (12.0)	61 (9.3)	526 (15.6)	47 (10.8)	
Other[d]	1614 (10.8)	1314 (11.4)	49 (12.4)	222 (9.1)	29 (7.4)	
**Education level**						<0.001
Lower than high school	1854 (13.3)	1278 (11.5)	84 (26.1)	470 (17.6)	22 (7.6)	
High school or equivalent	1884 (23.7)	1201 (19.5)	80 (27.8)	534 (34.8)	69 (42.1)	
Some college	2491 (30.4)	1720 (29.0)	120 (39.0)	556 (33.0)	95 (34.0)	
College graduate or above	2161 (32.6)	1905 (40.0)	18 (7.2)	210 (14.6)	28 (16.3)	
**Marital status**						<0.001
Married/living with partner	4966 (62.3)	3833 (66.4)	151 (56.7)	889 (53.3)	93 (37.2)	

Widowed/divorced/separated	1801 (17.9)	1233 (16.5)	65 (19.0)	473 (23.5)	30 (14.5)	
Never married	1628 (19.7)	1043 (17.2)	86 (24.3)	408 (23.2)	91 (48.4)	
**Family income**						<0.001
Low income	1624 (14.9)	992 (11.3)	82 (24.7)	514 (26.3)	36 (13.2)	
Middle income	3904 (48.5)	2802 (46.3)	153 (58.4)	837 (53.5)	112 (55.5)	
High income	1766 (36.5)	1507 (42.4)	32 (16.8)	188 (20.3)	39 (31.3)	
**BMI category**						0.113
Normal	2103 (27.7)	1477 (27.0)	76 (26.8)	490 (30.1)	60 (29.6)	
Overweight	2509 (31.1)	1864 (31.6)	86 (30.5)	509 (30.1)	50 (25.7)	
Obesity	3139 (39.5)	2317 (40.0)	116 (39.3)	626 (37.8)	80 (39.7)	
Underweight	138 (1.7)	76 (1.4)	8 (3.4)	45 (2.0)	9 (4.9)	
**Alcohol use**						0.063
No	1197 (15.4)	854 (15.3)	29 (11.5)	291 (17.6)	23 (10.0)	
Yes	4787 (84.6)	3162 (84.7)	235 (88.5)	1224 (82.4)	166 (90.0)	
**Sleep disorder**						<0.001
No	6239 (71.7)	4716 (74.8)	191 (60.9)	1183 (64.1)	149 (64.9)	
Yes	2157 (28.3)	1394 (25.2)	111 (39.1)	587 (35.9)	65 (35.1)	
**Diabetes**						0.199
No	6292 (87.6)	4522 (87.0)	239 (88.2)	1358 (88.7)	173 (92.5)	
Yes	1382 (12.4)	1069 (13.0)	33 (11.8)	262 (11.3)	18 (7.5)	
**Hypertension**						0.015
No	4201 (61.8)	3049 (62.3)	162 (58.4)	855 (58.4)	135 (75.0)	
Yes	3488 (38.2)	2514 (37.7)	119 (41.6)	796 (41.6)	59 (25.0)	

NHANES, National Health and Nutrition Examination Survey; SD, standard deviation; BMI, body mass index.

[a] Accounting for sampling weights.

[b] Calculated by Chi-square test or one-way analysis of variance.

[c] Individuals under 20 years old were not included in analyses due to a lack of information on nocturia and urge urinary incontinence.

[d] Other race includes American Indian or Alaska Native, Native Hawaiian or Pacific Islander, multiple races or ethnicities, or unknown.

Supplementary Table 3. Associations of cigarette use, e-cigarette use, and dual use of cigarettes and e-cigarettes with nocturia and urge urinary incontinence in males using data from the NHANES 2005-2020

Exposures	Nocturia, Odds ratio (95% CI)			Urge urinary incontinence, Odds ratio (95% CI)		
	**Model 1[a]**	**Model 2[b]**	**Model 3[c]**	**Model 1[a]**	**Model 2[b]**	**Model 3[c]**
**Cigarette use**						
Never use	ref	ref	ref	ref	ref	ref
Current use	1.15 (1.02-1.29)*	1.02 (0.90-1.16)	1.01 (0.89-1.14)	1.53 (1.29-1.82)***	1.39 (1.14-1.70)**	1.39 (1.12-1.74)**
Former use	1.24 (1.09-1.42)**	1.19 (1.04-1.37)*	1.15 (1.00-1.33)*	1.15 (1.00-1.32)*	1.08 (0.93-1.26)	1.01 (0.86-1.18)
**E-cigarette use**						
Never use	ref	ref	ref	ref	ref	ref
Current use	1.66 (1.13-2.42)*	1.75 (1.13-2.71)*	1.72 (1.07-2.76)*	1.58 (1.01-2.46)*	1.35 (0.81-2.25)	1.36 (0.83-2.23)
Former use	1.22 (0.92-1.62)	1.17 (0.86-1.59)	1.15 (0.84-1.58)	1.29 (0.83-2.00)	1.34 (0.86-2.08)	1.33 (0.86-2.07)
**Dual use of cigarettes and e-cigarettes**						
Never use	ref	ref	ref	ref	ref	ref
Dual use	1.72 (0.99-2.99)	1.74 (0.95-3.20)	1.74 (0.93-3.23)	1.65 (0.73-3.72)	1.30 (0.52-3.23)	1.47 (0.59-3.68)
Exclusive cigarette use	1.28 (0.95-1.72)	1.11 (0.78-1.57)	1.11 (0.78-1.57)	1.81 (1.29-2.53)**	1.68 (1.01-2.79)*	1.80 (1.01-3.22)*
Exclusive e-cigarette use	1.88 (1.06-3.33)*	1.90 (1.05-3.45)*	1.81 (0.92-3.54)	2.28 (1.19-4.38)*	2.16 (1.03-4.55)*	2.23 (1.00-4.99)

NHANES, National Health and Nutrition Examination Survey; CI, confidence interval; *** $p < 0.001$, ** $p < 0.01$, * $p < 0.05$; [a] Adjusted for age, race; [b] Adjusted for age, race, education level, marital status, and family income; [c] Adjusted for age, race, education level, marital status, family income, body mass index (BMI), alcohol use, and sleep disorders.

Supplementary Table 4. Associations of cigarette use, e-cigarette use, and dual use of cigarettes and e-cigarettes with nocturia and urge urinary incontinence in females using data from the NHANES 2005-2020

Exposures	Nocturia, Odds ratio (95% CI)			Urge urinary incontinence, Odds ratio (95% CI)		
	**Model 1[a]**	**Model 2[b]**	**Model 3[c]**	**Model 1[a]**	**Model 2[b]**	**Model 3[c]**
**Cigarette use**						
Never use	ref	ref	ref	ref	ref	ref
Current use	1.11 (0.97-1.26)	0.98 (0.86-1.13)	0.96 (0.84-1.11)	1.26 (1.11-1.44)***	1.11 (0.97-1.28)	1.10 (0.96-1.28)
Former use	1.14 (1.00-1.31)*	1.11 (0.96-1.27)	1.06 (0.91-1.23)	1.10 (0.98-1.24)	1.06 (0.94-1.20)	0.99 (0.87-1.13)
**E-cigarette use**						
Never use	ref	ref	ref	ref	ref	ref
Current use	1.24 (0.82-1.88)	1.09 (0.67-1.77)	1.06 (0.63-1.77)	1.90 (1.23-2.92)**	1.87 (1.26-2.78)**	1.78 (1.18-2.70)**
Former use	1.11 (0.88-1.42)	1.06 (0.85-1.33)	1.00 (0.80-1.24)	1.40 (1.11-1.77)**	1.25 (0.98-1.59)	1.18 (0.92-1.50)
**Dual use of cigarettes and e-cigarettes**						
Never use	ref	ref	ref	ref	ref	ref
Dual use	1.76 (0.95-3.29)	1.47 (0.76-2.84)	1.37 (0.69-2.72)	2.27 (1.34-3.83)**	2.24 (1.41-3.56)**	2.01 (1.16-3.48)*
Exclusive cigarette use	1.23 (0.89-1.70)	1.08 (0.78-1.50)	1.05 (0.76-1.45)	1.37 (1.04-1.80)*	1.24 (0.90-1.71)	1.22 (0.88-1.68)
Exclusive e-cigarette use	0.86 (0.50-1.48)	0.81 (0.38-1.73)	0.81 (0.36-1.84)	1.54 (0.72-3.29)	1.73 (0.80-3.76)	1.61 (0.75-3.43)

NHANES, National Health and Nutrition Examination Survey; CI, confidence interval; *** p <0.001, ** p <0.01, * p <0.05; [a] Adjusted for age, race; [b] Adjusted for age, race, education level, marital status, and family income; [c] Adjusted for age, race, education level, marital status, family income, body mass index (BMI), alcohol use, and sleep disorders.

STROBE Statement—Checklist of items that should be included in reports of ***cross-sectional studies***

	Item No	Recommendation	
**Title and abstract**	1	(*a*) Indicate the study's design with a commonly used term in the title or the abstract	Page 1
		(*b*) Provide in the abstract an informative and balanced summary of what was done and what was found	Page 1
**Introduction**			
Background/rationale	2	Explain the scientific background and rationale for the investigation being reported	Page 2-3
Objectives	3	State specific objectives, including any prespecified hypotheses	Page 3
**Methods**			
Study design	4	Present key elements of study design early in the paper	Page 3
Setting	5	Describe the setting, locations, and relevant dates, including periods of recruitment, exposure, follow-up, and data collection	Page 3
Participants	6	(*a*) Give the eligibility criteria, and the sources and methods of selection of participants	Page 3
Variables	7	Clearly define all outcomes, exposures, predictors, potential confounders, and effect modifiers. Give diagnostic criteria, if applicable	Page 3-6
Data sources/ measurement	8*	For each variable of interest, give sources of data and details of methods of assessment (measurement). Describe comparability of assessment methods if there is more than one group	Page 3-6
Bias	9	Describe any efforts to address potential sources of bias	Page 3
Study size	10	Explain how the study size was arrived at	Page 3
Quantitative variables	11	Explain how quantitative variables were handled in the analyses. If applicable, describe which groupings were chosen and why	Page 6
Statistical methods	12	(*a*) Describe all statistical methods, including those used to control for	Page 6

		confounding	
		(*b*) Describe any methods used to examine subgroups and interactions	Page 6
		(*c*) Explain how missing data were addressed	Page 6
		(*d*) If applicable, describe analytical methods taking account of sampling strategy	Page 6
		(*e*) Describe any sensitivity analyses	Page 6
**Results**			
Participants	13*	(a) Report numbers of individuals at each stage of study—eg numbers potentially eligible, examined for eligibility, confirmed eligible, included in the study, completing follow-up, and analysed	Page 6
		(b) Give reasons for non-participation at each stage	Page 6
		(c) Consider use of a flow diagram	Page 6
Descriptive data	14*	(a) Give characteristics of study participants (eg demographic, clinical, social) and information on exposures and potential confounders	Page 6-7
		(b) Indicate number of participants with missing data for each variable of interest	Page 6-7
Outcome data	15*	Report numbers of outcome events or summary measures	Page 6-7
Main results	16	(*a*) Give unadjusted estimates and, if applicable, confounder-adjusted estimates and their precision (eg, 95% confidence interval). Make clear which confounders were adjusted for and why they were included	Page 7-8
		(*b*) Report category boundaries when continuous variables were categorized	Page 7-8
		(*c*) If relevant, consider translating estimates of relative risk into absolute risk for a meaningful time period	Page 7-8
Other analyses	17	Report other analyses done—eg analyses of subgroups and interactions, and sensitivity analyses	Page 8-9
**Discussion**			
Key results	18	Summarise key results with reference to study objectives	Page 9

Limitations	19	Discuss limitations of the study, taking into account sources of potential bias or imprecision. Discuss both direction and magnitude of any potential bias	Page 10-11
Interpretation	20	Give a cautious overall interpretation of results considering objectives, limitations, multiplicity of analyses, results from similar studies, and other relevant evidence	Page 9-10
Generalisability	21	Discuss the generalisability (external validity) of the study results	Page 11
**Other information**			
Funding	22	Give the source of funding and the role of the funders for the present study and, if applicable, for the original study on which the present article is based	Page 11

*Give information separately for exposed and unexposed groups.

Note: An Explanation and Elaboration article discusses each checklist item and gives methodological background and published examples of transparent reporting. The STROBE checklist is best used in conjunction with this article (freely available on the Web sites of PLoS Medicine at http://www.plosmedicine.org/, Annals of Internal Medicine at http://www.annals.org/, and Epidemiology at http://www.epidem.com/). Information on the STROBE Initiative is available at www.strobe-statement.org.

© 2025 Yang X. et al.
